# Gene mutation discovery research of non-smoking lung cancer patients due to indoor radon exposure

**DOI:** 10.1186/s40557-016-0095-2

**Published:** 2016-03-16

**Authors:** Jung Ran Choi, Seong Yong Park, O Kyu Noh, Young Wha Koh, Dae Ryong Kang

**Affiliations:** Department of Bio-resource engineering, College of Life Sciences, Sejong University, Seoul, Republic of Korea; Department of Thoracic and Cardiovascular Surgery, Ajou University School of Medicine, Suwon, Republic of Korea; Department of Radiation Oncology, Ajou University School of Medicine, Suwon, Republic of Korea; Department of Pathology, Ajou University School of Medicine, Suwon, Republic of Korea; Department of Humanities and Social Medicine, Ajou University School of Medicine, Suwon, Republic of Korea

**Keywords:** Radon, Non-small-cell lung cancer, Nerver smoker, Genetic polymorphism, Next generation sequencing

## Abstract

Although the incidence and mortality for most cancers such as lung and colon are decreasing in several countries, they are increasing in several developed countries because of an unhealthy western lifestyles including smoking, physical inactivity and consumption of calorie-dense food. The incidences for lung and colon cancers in a few of these countries have already exceeded those in the United States and other western countries. Among them, lung cancer is the main cause of cancer death in worldwide. The cumulative survival rate at five years differs between 13 and 21 % in several countries. Although the most important risk factors are smoking for lung cancer, however, the increased incidence of lung cancer in never smokers(LCINS) is necessary to improve knowledge concerning other risk factors. Environmental factors and genetic susceptibility are also thought to contribute to lung cancer risk. Patients with lung adenocarcinoma who have never smoking frequently contain mutation within tyrosine kinase domain of the epidermal growth factor receptor(EGFR) gene. Also, K-ras mutations are more common in individuals with a history of smoking use and are related with resistance to EFGR-tyrosine kinase inhibitors. Recently, radon(Rn), natural and noble gas, has been recognized as second common reason of lung cancer. In this review, we aim to know whether residential radon is associated with an increased risk for developing lung cancer and regulated by several genetic polymorphisms.

## Background

Non-small cell lung cancer(NSCLC) is the leading cause of cancer-related death worldwide [1, 2]. During the past decades, survival rate of lung cancer has improved moderately and remains still poor (around 10 % at 5 years) [1–5]. Although early-stage lung cancer can be treated with good survival, most cases are diagnosed at a late stage when surgery as usual is no longer needed. Late-stage lung cancers observe poor response to chemotherapy and radiotherapy, although tyrosine kinase inhibitors were investigated to be efficient in reducing tumor tissue in NSCLC with epidermal growth factor receptor (EGFR) mutations. The most important risk factors are smoking for lung cancer, however, the increased incidence of lung cancer in non-smokers(LCINS) is necessary to concern other risk factors [3]. Recently, radon(Rn), natural and noble gas, has been recognized as second common reason of lung cancer. Still, smoking is related to lung cancer risk factor, this review focused on NSCLC in never smokers.

## Lung cancer in never smoker

Smoking explains for more than 90 % of lung cancers in men and 75 to 85 % lung cancers in women in the United States and European Union. Although these patterns are similar in Asian men, the proportion of Asian women with lung cancer in smoker is much lower. The proportion of women with lung cancer who have smoking differs from region even within Asia, from 25 % in Korea to 56 % in Hong Kong [6]. Tobacco smoking still remains the predominant risk factor for the development of lung cancer. However, lung cancer induces also in individuals without a history of smoking [7–9].

LCINS has been acknowledged as a disease entity separated from smoking-associated lung cancer [10]. According to the World Health Organization, the incidence of LCINS is almost 25 % of all cases [8, 11]. Recently, there is noticeable variance in the ratios of LCINS ranging from nearly 10 % in males in Western and up to approximately 40 % in females in Asia [7]. However, its ethnic/genetic attributes and/or environmental features remain still unknown.

LCINS occurs more often in women than in men and the major histological subtype is adenocarcinoma [10]. During decades, the incidence of lung adenocarcinoma has increased compared to that of squamous cell carcinoma in western and Asian countries [12]. Small-cell lung cancer is rarely investigated in never-smokes, while in NSCLC, the most common histological type in never smokers is adenocarcinoma [7, 11, 13, 14]. Toh CK et al. [13] shown adenocarcinomas involved 69.9 % of patients in never smokers, 39.9 % in current and 47.3 % in former smokers [13]. Especially in Asian countries, a significant proportion of current patients with lung cancer are never smoker. It shown 38.3 % of 10,279 patients with smoking status in Japan, 32.4 % of 883 patients in Singapore, and 34.5 % of 4622 patients in Korea were never smokers [12].

Numerous studies have been demonstrated the roles of candidate susceptibility in LCINS and those was involved in carcinogen metabolism, DNA repair or inflammatory response. However, their role remains still to be identified and further studies are needed to better understand the role for genetic factors in LCINS development and treatment [7]. Recently, it has been observed LCINS has a dramatic response to EGFR tyrosine kinase inhibitors (TKIs) [15, 16]. The distinct response pattern was associated with the higher frequencies of EGFR mutations in never smokers [10, 15, 16].

Several studies have shown LCINS patients have better survival compared to ever smoker patients [17, 18]. The biologic dissimilarity of lung cancer in never-smokers versus ever-smokers are shown in differential response according to specific therapies including EGFR inhibitors, and in distribution of histology such as adenocarcinoma in never-smokers [11]. Therefore, better understanding of the incidence rate and etiology of LCINS is important because of the implications for therapeutic trials and epidemiologic studies of lung cancer [7].

## Genetic susceptibility and LCINS

Despite standard platinum-based chemotherapy and newer targeted therapies, there is an increasing need for the appropriate use of targeted and tailored therapies to improve efficiency and response in LCINS [17, 18]. In an attempt to improve the outcomes of the therapy, the role of inherited genetic factors in the development of non-smoking related carcinogenesis is investigated (Table 1).

**Table 1:** Genetic susceptibility and lung cancer in nerver smokers

Author/year	Region	No. of pts.	Histologic type	Genetic markers
McKay JD et al. 2008 [20]	France	3259 case	adenocarcinoma	CLPTM1L-TERT
		4159 control		
Hsiung CA et al. 2010 [21]	Taiwan	584 case	adenocarcinoma	CLPTM1L-TERT
		585 control		
Iwamoto S et al. 2014 [22]	Japan	341	NSCLC	EPAS1
Kang HG et al. 2014 [23]	Korea	360	lung cancer	CSF1R, TP63, CIR1
Shen L et al. 2014 [24]	China	1003	adenocarcinoma	ATM
Yongjun Zhang MM et al. 2013 [25]	China	400	NSCLC	TGM5, PPAP2B, PSMA4
Sun Z et al. 2014 [26]	USA	27	adenocarcinoma	EGFR, TP53, KRAS, RPS6KB2, ATXN2, DHX9, PTPN13, SP1, SPTAN1, MYOF
Bennett WP et al. 1999 [27]	USA	106	lung cancer	GSTM1
Ahn MJ et al. 2012 [28]	Korea	446	NSCLC	APCDD1, NAPG, FAM38B
Lim WY et al. 2011 [29]	Singapore	433		IL6, cyclooxygenase-2, PPAR-γ, IL1RN
Li Y et al. 2010 [30]	USA	1489	lung cancer	13q31.3 GPC5
Wu X et al. 2013 [31]	USA	1583	NSCLC	LEMD3, TMBIM, ATXN7L2, SHE, ITIH2, NUDT5
Zhou W et al. 2003 [32]	USA	1091	lung cancer	XRCC1, ERCC2
Hung RJ et al. 2003 [33]	France	302 case	lung cancer	CYP1A1, GSTM1
		1631 control		
Liu L et al. 2014 [34]	China	298 case	lung cancer	GPC5
		599 control		

Recently, genome-wide association(GWAS) studies identified specific chromosomal locus, 15q24-15q25 and 5p15.33, as one of the regions associated with LCINS. This 5p15.33 region consists two candidate susceptibility genes, TERT and CLPTMIL and Hsiung study included only lung adenocarcinoma never smoker female [19–21].

Hypoxia-inducible factor-2α (also called endothelial periodic acid-Schiff domain protein 1, EPAS1) seems to play an important role in some carcinogenesis, though there is no information on the relationship between single nucleotide polymorphism(SNP) of EPAS1 and lung cancer development so far [22]. Iwamoto S et al. observed EPAS1 rs4953354 may be a potentially susceptible marker for development of lung adenocarcinoma, especially in female never-smokers [22].

Kang HG et al. [23] identified three SNPs (colony-stimulating factor 1 receptor, CSF1R; tumor protein p63, TP63; and corepressor interacting with RBPJ 1, CIR1) were found to be significantly associated with lung cancer. Among them, Kang HG et al. suggested CSF1R rs10079250 may contribute to lung cancer susceptibility in never-smoking females [23].

The ataxia-telangiectasia mutated (ATM) gene plays a crucial role in the DNA double-strand breaks repair pathway. Shen L et al. [24] suggested ATM rs189037 might be associated with the risk of lung adenocarcinoma in Chinese non-smoking females. Furthermore, ATM rs189037 AA genotype might be a risk factor of lung adenocarcinoma among female non-smokers without cooking oil fume exposure [24].

Yongjun Zhang MM et al. investigated the potential association between SNPs in transglutaminase 5 (TGM5), phosphatidic acid phosphatase type 2B (PPAP2B) and proteasome subunit, alpha type 4 (PSMA4) and NSCLC susceptibility in Chinese patients who were non-smokers [25]. The polymorphisms of TGM5, PPAP2B and PSMA4 are not major contributors to NSCLC susceptibility, however, this primarily be attributed to the significantly distinct genetic background of Asian populations from western populations [25].

Until now, several reports have been studied the role of candidate genetic polymorphisms in LCINS (Table 1), [26–35] however, their role remains still to be identified and further studies are needed to better understand the role for genetic factors in LCINS developing.

## Radon as one of risk factors for LCINS

Although the most established risk factor for lung cancer is smoking in several studies, unrelated to smoking risk factors such as second hand smoking, radon exposure, cooking oil vapors and indoor coal burning, hormonal factors, and infectious factors have been identified [7]. Among them, radon (Rn) is the most important natural source of human exposure to ionizing radiation and the second leading cause of LCINS [36, 37]. The Environmental Protection Agency (EPA) action level is 148 Bq/m^3^ and the World Health Organization (WHO) has recently lowered the recommended radon exposure to levels below 100 Bq/m^3^ [38]. Many case–control studies have presented the association between airborne radon exposure and lung cancer, however, several observations have analyzed the effect of radon exposure for different categories of smoking [38]. On the other hand, possible biologic mechanisms by radon exposure might increase the risk of LCINS leading gene mutation, chromosome aberrations, generation of reactive oxygen species, up or down-regulation of cytokines, and production of proteins associated with cell cycle regulation [36].

## Radon exposure and genetic factors

Sinitsky MY et al. suggested the elevated frequency of cytogenetic damage in people with DNA-repair gene polymorphisms due to chronic exposure to radon and XpG, ADPRT, and NBS1 gene can be used as molecular genetic markers of increased individual radiosensitivity in long-term residents with high concentrations of radon [39]. Another report suggested GSTM1 and GSTT1 genes deletion increase the risk of lung cancer and these genes might regulate the carcinogenic pathway by radon radiation [40, 41]. Torres-Durán M et al. shown radon exposure of never smokers seems to be a risk factor for lung cancer and individuals diagnosed at a younger age might have a higher residential radon concentration explaining an accumulative effect on lung cancer appearance (Table 2) [42, 43].

**Table 2:** Genetic susceptibility in subjects with high radon concentration

Author/year	Region	Subjects	Gene
Sinitsky MY et al. 2015 [39]	Russia	children	ADPRT, hOGG1, NBS1, XRCC1, XpC, XpD, XpG
Ruano-Ravina A et al. 2014 [40]	Spain	792 lung cancer	GSTM1,GSTT1
Bonner MR et al. 2006 [41]	US	270 lung cancer	GSTM1
Druzhinin VG et al. 2011 [44]	Russia	healthy voluteers	hOGG1, ADPRT, APE1, XRCC1, XpG, XpC, XpD, NBS1
Kiuru A et al. 2005 [45]	Finland	84 healthy nonsmokers	OGG1, XPD, XRCC1, XRCC3
Yngveson A et al. 1999 [46]	Sweden	83 nonsmoking lung cancer	p53
		250 smoking lung cancer	
Hayes VM et al. 1996 [47]	South Africa	SCLC	TP53
Takeshima Y et al. 1996 [48]	Japan	28 adenocarcinomas non smoking female	p16/CDKN2, p53
Leng S et al. 2013 [49]	USA	267 SCC case	SIRT1
		383 control	
Leng S et al. 2015 [50]	USA	242 SCC	IL-6
		336 control	

Although, several studies have been demonstrated the role of candidate genes for developing LCINS, however, the genetic determinants for susceptibility in LCINS with residential radon exposure are still uncertain (Table 2), [44–50] and further studies will require analyzing the association between radon exposure and LCINS.

## Next-generation sequencing (NGS) for analyzing of SNP in LCINS

Next-generation sequencing (NGS) is a cost-effective technology enabled screening several genes simultaneously, however, its application in a clinical context needs an established workflow to acquire available sequencing results. When combined with various selective capture approaches, NGS has allowed for the efficient simultaneous genetic analysis of a large number of candidate genes. Several studies applied a polymerase chain reaction (PCR) based NGS in determining oncogene alternations in the state of disease progression [51–53]. PCR based NGS is an outstanding tool to provide a comprehensive genomic diagnosis in patients with recurrent LCINS [53]. Recently, Vanni I et al. [51] investigated an optimized NGS workflow analyzing 22 lung cancer-related genes to sequence critical samples such as DNA from formalin-fixed paraffin-embedded (FFPE) blocks [51]. Masago K et al. [52] shown NGS is able to detect EGFR T790M mutations in cases not easy to diagnose by other conventional methods and play a role in acquired EGFR-TKIs resistance, showing the need for alternative treatment strategies, with PCR-based NGS playing an important role in disease diagnosis.

**Fig. 1:**
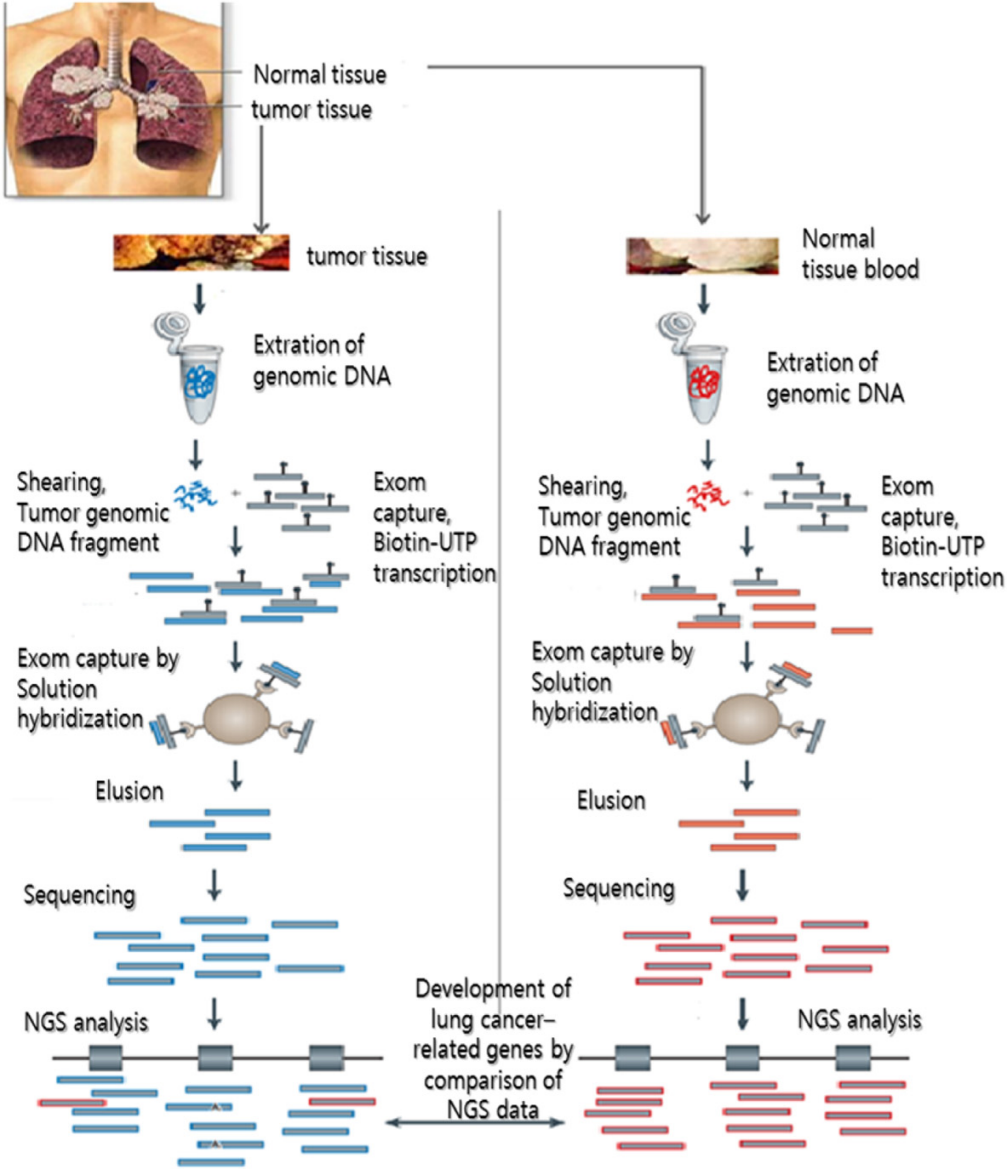
Strategy for detecting lung cancer-related genes using exom sequencing based on NGS in LCINS tissues

Here, we suggest the strategy based on NGS enabled to detect lung cancer-related genetic mutations in LCINS patients with radon exposure (Fig. 1). We will optimize exom sequencing based on NGS platform to analyze LCINS-related gene in normal and lung cancer tissues.

## Conclusion

Several etiologic factors have been proposed for the development of LCINS, including exposure to radon, cooking fumes, asbestos, heavy metals, and environmental tobacco smoke, human papillomavirus infection, and inherited genetic susceptibility. However, the relative significance of radon exposure and genetic polymorphisms in the development of LCINS has not been well-characterized. This review summarized whether radon exposure is associated with an increased risk of developing lung cancer and regulated by several genetic polymorphisms in never smokers.
